# Glucocorticoid Resistance is Associated with Poor Functional Outcome After Stroke

**DOI:** 10.1007/s10571-020-00818-1

**Published:** 2020-02-27

**Authors:** Anna Maria Lopatkiewicz, Elzbieta Gradek-Kwinta, Mateusz Czyzycki, Joanna Pera, Agnieszka Slowik, Tomasz Dziedzic

**Affiliations:** Department of Neurology, Jagiellonian University Medical College, ul. Botaniczna 3, 31-503 Kraków, Poland

**Keywords:** Stroke, Outcome, Inflammation, Glucocorticoid sensitivity

## Abstract

Systemic inflammation is associated with poor outcome after stroke. Glucocorticoids (GCs) play a fundamental role in limiting inflammation. The aim of this study was to explore the associations between GC sensitivity, systemic inflammation, and outcome after ischemic stroke. The study population compised 246 ischemic stroke patients (median age: 69.0 years; 41.1% female). To assess GC sensitivity, we incubated venous blood samples that were obtained at day 3 after stroke with lipopolysaccharide (10 ng/mL) and dexamethasone (10^–6^ mol/L). We defined the GC sensitivity index as the ratio of tumor necrosis factor α (TNFα) released after blood stimulation with lipopolysaccharide and dexamethasone to the amount of TNFα released after blood stimulation with lipopolysaccharide alone. A higher index indicates higher GC resistance. The patients with poor functional outcome had a higher GC sensitivity index than those with good outcome (median: 16.1% vs. 13.5%, *P* < 0.01). In a logistic regression analysis adjusted for age, stroke severity, pneumonia, leukocyte count, plasma interleukin-6, and TNFα release ex vivo, a higher GC sensitivity index was associated with a higher risk of poor outcome after stroke (OR 2.32, 95% CI 1.21–4.45, *P* = 0.01). In conclusion, GC resistance is associated with poor functional outcome after stroke.

## Background

Acute stroke is accompanied by both systemic inflammation and immunodepression (Chamorro et al. 2012; Murray et al. 2013). Systemic inflammation is reflected by an elevated level of circulating interleukin-6 (IL-6) and C-reactive protein (CRP) (Dziedzic 2015). Immunodepression manifests as a functional deactivation of monocytes, lymphopenia, and spleen atrophy (Chamorro et al. 2012). The laboratory hallmark of immunodepression is a reduction in tumor necrosis factor α (TNFα) release after blood stimulation ex vivo with endotoxin. Elevated levels of circulating IL-6 and CRP as well as reduced TNFα production ex vivo are associated with poor outcome after stroke (Chamorro et al. 2012; Dziedzic 2015; Klimiec et al. 2018).

Glucocorticoids (GCs) are an important element of the feedback mechanism in the immune system and restrain the immune response. A failure of GCs to inhibit the inflammatory reaction may contribute to disease development. The ultimate biological response to GCs is determined by the concentration of GCs and individual differences in GC sensitivity (Silverman and Sternberg 2012; Quax et al. 2013).

Multiple factors that act at the level of GC receptors and their signaling pathway can influence the response to GCs. Among them, inflammation may contribute to GC resistance (Pace et al. 2007; Pace and Miller 2009). Cytokines such as TNFα, IL-1, and IL-6 can decrease the expression of GC receptors, inhibit translocation of the GC receptors from the cytoplasm to the nucleus, and disrupt the protein–protein interactions of GC receptors and their binding to DNA. Moreover, cytokines can induce relatively inert receptor isoforms that exhibit reduced binding affinity.

Another way in which cytokines might contribute to GC resistance is the regulation of GC bioavailability. Proinflammatory cytokines tend to favor increased GC bioavailability by reducing levels of corticosteroid binding globulin (CBG) and multidrug-resistant P-glycoprotein (MDR). CBG binds to a majority of circulating cortisol and limits its activity because only unbound cortisol is able to diffuse across cell membranes and interact with GC receptors. MDR is an efflux pump that decreases the intracellular concentrations of GCs. In addition, cytokines can influence the expression of 11-β-hydroxysteroid dehydrogenase isoenzymes, which play an important role in the regulation of local concentrations of cortisol.

In animal studies, increased activity of GC-inducible kinase 1 exacerbated ischemic brain damage (Inoue et al. 2016). Moreover, the proteasome-dependent degradation of GC receptors contributes to GC insensitivity at the hypoxic blood–brain barrier (Kleinschnitz et al. 2011).

The aim of this study was to explore the associations between GC sensitivity, circulating IL-6 as a marker of systemic inflammation, and outcome after ischemic stroke. We hypothesized that GC resistance is associated with poor functional outcome after stroke.

## Methods

### Patient Selection and Clinical Assessment

The participants in this study were prospectively recruited from consecutive stroke patients who were hospitalized in the Department of Neurology, University Hospital, Krakow, Poland, between October 2016 and October 2018. The inclusion criteria were: (1) ischemic stroke; (2) time from the onset of stroke symptoms to admission < 24 h; (3) pre-stroke-modified Rankin Scale (mRS) score of 0–2 (independent of daily activities); (4) National Institute of Health Stroke Scale (NIHSS) score on admission > 3; and (5) informed patient consent. The exclusion criteria were: (1) chronic inflammatory, autoimmune, or cancerous diseases; and (2) the use of steroids or immunomodulatory drugs before stroke or in the acute phase of stroke. Written informed consent was obtained from each patient included in the study. The study protocol was approved by the Bioethics Committee of Jagiellonian University.

The neurological deficit on admission was assessed using the NIHSS, which quantifies stroke-related neurological deficit. Higher scores indicate greater impairment and more severe stroke (Lyden et al. 1994).

Functional outcome was assessed at 3 months after stroke using mRS which is a 7-grade scale used to measure the degree of disability. Scores of 0 to 2 indicate functional independence, scores of 3 to 5 indicate dependence of the patient in daily activities, and 6 indicates death (van Swieten et al. 1998). Unfavorable outcomes were defined as a mRS of 3 to 6.

Stroke etiology was determined using the TOAST criteria (Adams et al. 1993).

### Laboratory Assays

Venous blood was collected in heparinized tubes (Sarstedt, Germany) at day 3 after stroke. To avoid diurnal variation, the blood was obtained between 7:00 and 7:30 AM. The whole blood was diluted by 1:5 in sterile RPMI 1640 medium supplemented with l-glutamine (Sigma-Aldrich, St. Louis, MO). The samples were then stimulated in sterile tubes (Lonza, Walkersville, MD) for 4 h at 37 °C in 5% CO_2_ with LPS (10 ng/mL, *Escherichia coli* 0111:B4, Sigma-Aldrich, St. Louis, MO) or LPS and dexamethasone-21-phosphate (10^–6^ mol/L, Sigma-Aldrich, St. Louis, MO). The supernatants were removed and stored at − 80 °C until further analysis.

Similarly to previous studies, TNFα was chosen as an indicator of GC sensitivity because this cytokine has the greatest sensitivity to GCs in comparison to IL-1β and IL-6 (DeRijk et al. 1997).

The GC sensitivity index was defined as the ratio of TNFα released after blood stimulation with LPS and dexamethasone to the amount of TNFα released after blood stimulation with LPS alone. A higher index indicates lower GC sensitivity (and higher GC resistance).

TNFα and IL-6 concentrations were measured using a commercially available ELISA kit (R&D Systems, Minneapolis, MN) according to the manufacturer’s instructions. The cytokine detection limits were 0.19 pg/mL for TNFα and 0.11 pg/mL for IL-6. For both cytokines, the intra-assay CVs were < 5%, and the inter-assay CVs were < 10%.

### Statistical Analysis

The *χ*^2^ test was used to compare proportions, while the Mann–Whitney *U* test and Kruskal–Wallis test were used to compare continuous variables between groups. Logistic regression was used to determine the predictors of functional outcome. Patients within the upper tertile of the GC sensitivity index were compared to the patients in other tertiles. The receiver operating characteristic curves were used to find an optimal cutoff level of GC sensitivity index that differentiates patients with good outcome from patients with poor outcome. The variables with *P* ≤ 0.05 in the univariate analysis were included in the multivariate analysis. The calculations were performed using the program STATISTICA for Windows (version 12.5, Statsoft, Poland).

## Results

We initially included 255 patients (median age: 69.0 years; 41.4% female; median NIHSS: 10). However, information about outcome was not available for 9 patients. Thus, the final cohort included 246 patients (median age: 69.0 years; 41.1% female; median NIHSS: 9).

The baseline characteristics of the included patients categorized by tertiles of the GC sensitivity index are shown in Table 1.

**Table 1:** The baseline characteristics of patients categorized by tertiles of the GC index

	Lower tertile (*N* = 81)	Middle tertile (*N* = 82)	Upper tertile (*N* = 83)	*P* value
Age, median (IQs)	66 (57–77)	69 (63–77)	71 (63–81)	0.07
Female, *n* (%)	33 (40.7)	31 (37.8)	37 (44.6)	0.67
Hypertension, *n* (%)	59 (72.8)	68 (82.9)	66 (79.5)	0.28
Diabetes mellitus, *n* (%)	26 (32.1)	19 (23.2)	23 (27.7)	0.44
Atrial fibrillation, *n* (%)	22 (27.2)	26 (31.7)	23 (27.7)	0.78
Myocardial infarction, *n* (%)	8 (9.9)	10 (12.2)	15 (18.1)	0.29
Previous stroke or transient ischemic attack, *n* (%)	8 (9.9)	8 (9.8)	13 (15.7)	0.41
NIHSS score on admission, *n* (%)	7 (4–16)	8.5 (5–16)	13 (6–18)	** < 0.01**
Stroke etiology				0.17
Large vessel disease, *n* (%)	21 (25.9)	20 (24.4)	24 (28.9)	
Small vessel disease, *n* (%)	5 (6.2)	7 (8.5)	1 (1.2)	
Cardio-embolic, *n* (%)	21 (25.9)	30 (36.6)	22 (26.5)	
Other, *n* (%)	29 (35.8)	24 (29.3)	34 (41.0)	
Undermined, *n* (%)	5 (6.2)	1 (1.2)	2 (2.4)	
In-hospital pneumonia, *n* (%)	0 (0)	1 (1.2)	8 (9.6)	** < 0.01**
Intravenous thrombolysis, *n* (%)	47 (58.0)	46 (56.1)	45 (54.2)	0.89
Mechanical thrombectomy, *n* (%)	24 (29.6)	22 (26.8)	19 (22.9)	0.62
White blood cells count, × 10^3^/µL, median (IQs)	7.7 (6.5–9.3)	8.1 (6.5–9.9)	9.3 (7.7–11.4)	** < 0.01**
Serum IL-6 (pg/mL), median (IQs)	3.5 (1.8–8.0)	4.9 (2.3–10.9)	6.9 (3.1–21.1)	** < 0.01**
Ex vivo TNFα release after LPS stimulation (pg/mL), median (IQs)	2689 (1903–3826)	2268 (1725–3451)	2060 (1517–2792)	**0.04**
Poor outcome, *n* (%),	24 (29.6)	32 (39.0)	54 (65.1)	** < 0.01**

Statistically significant *P* values are highlighted in bold

*GC* glucocorticoid, *IQ* interquartile, *NIHSS* National Institute of Health Stroke Scale

The NIHSS score, white blood cell (WBC) count, rate of pneumonia, and plasma level of IL-6 increased across the tertiles of the GC sensitivity index whereas the TNFα release ex vivo decreased. The frequency of poor outcome was the lowest in patients in the lower tertile and the highest in patients in the upper tertile of the GC sensitivity index.

The baseline characteristic of patients with good outcome and patients with poor outcome are shown in Table 2.

**Table 2:** The baseline characteristics of patients with good outcome and patients with poor outcome 3 months after stroke

	Good outcome (*N* = 136)	Poor outcome (*N* = 110)	*P* value
Age, median (IQs)	66 (58–75.5)	72 (63–81)	** < 0.01**
Female, *n* (%)	52 (38.2)	49 (48.5)	0.32
Hypertension, *n* (%)	103 (75.7)	90 (81.8)	0.25
Diabetes mellitus, *n* (%)	34 (25.0)	34 (30.9)	0.30
Atrial fibrillation, *n* (%)	40 (29.4)	31 (28.2)	0.83
Myocardial infarction, *n* (%)	17 (12.5)	16 (14.5)	0.64
Previous stroke or transient ischemic attack, *n* (%)	13 (9.6)	16 (14.5)	0.23
NIHSS score on admission, *n* (%)	6.5 (4–13)	14.5 (7–19)	** < 0.01**
Stroke etiology			0.14
Large vessel disease, *n* (%)	28 (20.6)	37 (33.6)	
Small vessel disease, *n* (%)	9 (6.6)	4 (3.6)	
Cardio-embolic, *n* (%)	44 (32.3)	29 (26.4)	
Other, *n* (%)	6 (4.4)	2 (1.8)	
Undermined, *n* (%)	49 (36.0)	38 (34.5)	
In-hospital pneumonia, *n* (%)	1 (0.7)	8 (7.3)	** < 0.01**
Intravenous thrombolysis, *n* (%)	77 (56.6)	61 (55.4)	0.85
Mechanical thrombectomy, *n* (%)	39 (28.7)	26 (40.0)	0.37
White blood cells count, × 10^3^/µL, median (IQs)	7.7 (6.5–9.0)	9.5 (7.7–11.9)	** < 0.01**
Serum IL-6 (pg/mL), median (IQs)	3.2 (1.8–6.4)	8.7 (4.6–24.3)	** < 0.01**
Ex vivo TNFα release after LPS stimulation (pg/mL), median (IQs)	2607 (1774–3809)	2089 (1517–2835)	** < 0.01**
GC sensitivity index (%), median (IQs)	13.5 (11.4–16.0)	16.1 (13.7–19.7)	** < 0.01**

Statistically significant *P* values are highlighted in bold

*GC* glucocorticoid, *IQ* interquartile, *NIHSS* National Institute of Health Stroke Scale

The patients with poor outcome were older, had more severe neurological deficit on admission, and more frequently suffered from in-hospital pneumonia. Compared to the patients with good outcome, the patients with poor outcome had higher plasma IL-6 levels and lower TNFα release after blood stimulation with LPS.

The GC index was higher in patients with poor outcome. The GC index correlated with plasma IL-6 levels (*R* = 0.21, *P* < 0.05) and TNFα release ex vivo (*R* = − 0.16, *P* < 0.05).

The odds ratio (OR) for the highest tertile versus the other tertiles of the GC index was 3.56 (95% CI 2.04–6.21, *P* < 0.01) for poor functional outcome. Other predictors of poor outcome were: age (OR 1.04, 95% CI 1.02–1.06, *P* < 0.01), NIHSS score (OR 1.11, 95% CI 1.07–1.16, *P* < 0.01), pneumonia (OR 10.59, 95% CI 1.29–86.92, *P* = 0.03), WBC count (OR 1.31, 95% CI 1.17–1.46, *P* < 0.01), plasma IL-6 (OR 1.04, 95% CI 1.02–1.06, *P* < 0.01) and TNFα release ex vivo (OR 0.73, 95% CI 0.59–0.89, *P* < 0.01). After adjusting for age, NIHSS score, pneumonia, WBC count, plasma IL-6, and TNFα release, the OR for poor functional outcome was 2.32 (95% CI 1.21–4.45, *P* = 0.01).

In the whole group of patients, a GC index above 13.7% was associated with poor outcome both the univariate analysis (OR 3.53, 95% CI 2.02–6.16, *P* < 0.01) and the multivariate analysis adjusted for age, NIHSS score, pneumonia, WBC count, plasma IL-6, and TNFα release (OR 2.16, 95% CI 1.15–4.05, *P* = 0.02).

The results of one study suggested that diabetic patients might have blunted GC sensitivity to stress (Carvalho et al. 2015). After the exclusion of diabetic patients, the GC sensitivity index remained an independent predictor of poor outcome in the multivariate analysis (OR 2.55, 95% CI 1.19–5.46, *P* = 0.01).

## Discussion

We found that GC resistance was associated with poor functional outcome after stroke. This association was independent of not only age and stroke severity, which are the most important stroke prognosticators, but also from circulating IL-6, a marker of systemic inflammation, and ex vivo release of TNFα, a marker of immunodepression (Chamorro et al. 2012). To the best of our knowledge, this is the first study to investigate the relationship between GC resistance and outcome in stroke patients.

Our study revealed an association between GC sensitivity, circulating IL-6 and, to a lesser degree, TNFα synthesized ex vivo. The plasma level of IL-6 increased with the tertiles of the GC sensitivity index, whereas TNFα release ex vivo decreased. There are several hypothetical possibilities regarding the relationship between GC resistance, systemic inflammation, and post-stroke immunodepression. First, circulating cytokines including IL-6 could trigger GC resistance (Pace et al. 2007; Pace and Miller 2009). Second, GC resistance could lead to systemic inflammation by inadequate control of the immune reaction by GCs. Third, excessive GC release after cerebral ischemia might contribute to immunodepression (Mracsko et al. 2014). Fourth, since plasma IL-6 levels, TNF release ex vivo, and GC resistance depend on stroke severity, one common biological mechanism induced by brain injury could be responsible for all these phenomena.

The exact biological pathways leading to GC resistance in patients with poor outcome remain unknown. GC sensitivity is modulated by numerous genetic and acquired factor, including inflammatory mediators. Further studies are needed to explore the potential mechanisms of GC resistance in acute stroke. Better insight into the pathogenesis of GC resistance in stroke patients might be obtained through measurements of diurnal cortisol release, cortisol bioavailability, the expression of GC receptors in different populations of blood cells, GC receptor affinity, and GC receptor post-translational modification.

GCs regulate many physiological processes beyond the immune system. They can exert a modulatory effect on a variety of brain functions, including neurotransmission and neuronal plasticity (Gray et al. 2017). Thus, GC resistance might have a negative impact on stroke outcome independently from impaired counter-regulatory control of the immune response. An example of GC action beyond the immune system is the stabilization of the blood–brain barrier and the mitigation of cerebral edema. Kleinschnitz et al. (2011) showed GC insensitivity at the level of the blood–brain barrier in mice subjected to cerebral ischemia. This GC resistance may facilitate brain edema and consequently lead to worse outcome.

Pharmacological modulation of GC sensitivity could be considered as a potential strategy to improve stroke outcome. GC resistance is largely caused by the de-activation of histone deacetylase 2 (HDAC2), which is critical for the activity of GC receptors that mediate the anti-inflammatory effect (Barnes 2011). Selective activation of HDAC2 can be achieved with theophylline, which restores HDAC2 activity in macrophages and reverses GC resistance (Cosio et al. 2004). Further studies are needed to determine whether the pharmacological modulation of GC sensitivity in addition to reperfusion therapy is beneficial in stroke.

Our work has several limitations. We measured the GC index only once and used only one dose of dexamethasone to assess GC sensitivity. We took blood samples at day 3 after stroke rather than upon admission. However, this minimized the diurnal variation in cytokine production related to the time of blood collection. Furthermore, it allowed us to grasp the effect of systemic inflammation because the blood IL-6 level rises between 6 and 72 h after the onset of stroke symptoms (Pusch et al. 2015). Finally, our observational study is unable to demonstrate a causal relationship between GC resistance and stroke outcome.

## Conclusions

GC resistance is associated with poor functional outcome after stroke. These novel clinical observations might be important for future experimental studies exploring the relationship between GCs, inflammation, and stroke prognosis.
